# Recent Advancements in AAV-Vectored Immunoprophylaxis in the Nonhuman Primate Model

**DOI:** 10.3390/biomedicines11082223

**Published:** 2023-08-08

**Authors:** Elena S. B. Campbell, Melanie M. Goens, Wenguang Cao, Brad Thompson, Leonardo Susta, Logan Banadyga, Sarah K. Wootton

**Affiliations:** 1 Department of Pathobiology, University of Guelph, Guelph, ON N1G 2W1, Canada; 2 Public Health Agency of Canada, Winnipeg, MB R3E 3R2, Canada; 3 Avamab Pharma Inc., Calgary, AB T2T 2P9, Canada

**Keywords:** adeno-associated virus (AAV) vectors, therapeutic monoclonal antibodies, vectored immunoprophylaxis (VIP), nonhuman primate (NHP) model, human immunodeficiency virus (HIV), passive immunization, infectious diseases

## Abstract

Monoclonal antibodies (mAbs) are important treatment modalities for preventing and treating infectious diseases, especially for those lacking prophylactic vaccines or effective therapies. Recent advances in mAb gene cloning from naturally infected or immunized individuals has led to the development of highly potent human mAbs against a wide range of human and animal pathogens. While effective, the serum half-lives of mAbs are quite variable, with single administrations usually resulting in short-term protection, requiring repeated doses to maintain therapeutic concentrations for extended periods of time. Moreover, due to their limited time in circulation, mAb therapies are rarely given prophylactically; instead, they are generally administered therapeutically after the onset of symptoms, thus preventing mortality, but not morbidity. Adeno-associated virus (AAV) vectors have an established record of high-efficiency in vivo gene transfer in a variety of animal models and humans. When delivered to post-mitotic tissues such as skeletal muscle, brain, and heart, or to organs in which cells turn over slowly, such as the liver and lungs, AAV vector genomes assume the form of episomal concatemers that direct transgene expression, often for the lifetime of the cell. Based on these attributes, many research groups have explored AAV-vectored delivery of highly potent mAb genes as a strategy to enable long-term expression of therapeutic mAbs directly in vivo following intramuscular or intranasal administration. However, clinical trials in humans and studies in nonhuman primates (NHPs) indicate that while AAVs are a powerful and promising platform for vectored immunoprophylaxis (VIP), further optimization is needed to decrease anti-drug antibody (ADA) and anti-capsid antibody responses, ultimately leading to increased serum transgene expression levels and improved therapeutic efficacy. The following review will summarize the current landscape of AAV VIP in NHP models, with an emphasis on vector and transgene design as well as general delivery system optimization. In addition, major obstacles to AAV VIP, along with implications for clinical translation, will be discussed.

## 1. Introduction

Infectious diseases have had a profound impact on human health, in some cases with devastating and long-lasting consequences for a country’s economy. Despite the notable medical advancements of the past few decades, the recurrent emergence and re-emergence of infectious diseases, as well as the continued high disease burden of low- and middle-income countries, poses a significant threat from both the global public health and economic standpoints. Moreover, the development of effective vaccines for some of the world’s most important infectious diseases, such as human immunodeficiency virus (HIV), Zika virus, Cytomegalovirus, and Chikungunya virus remains elusive [1].

Recent technical progress in monoclonal antibody (mAb) isolation and production technologies has influenced a shift in focus towards passive immunization strategies for challenging vaccine targets [2]. Rather than stimulating the host immune system via immunogens to generate protective antibodies, passive immunization relies on the delivery of these proteins as prophylactics [3]. This is an attractive approach, particularly for the immunocompromised population, because it induces rapid immune protection in a manner that is largely independent of the host immune system. Additionally, this approach can provide protection against multiple serotypes of a particular pathogen and has similar efficacy and tolerability to many active immunization approaches [2]. While effective, the main caveat to traditional passive immunization is that protection is transient and repeated infusions are required. From an economic standpoint, this is not feasible or sustainable on a global scale.

**Figure 1:**
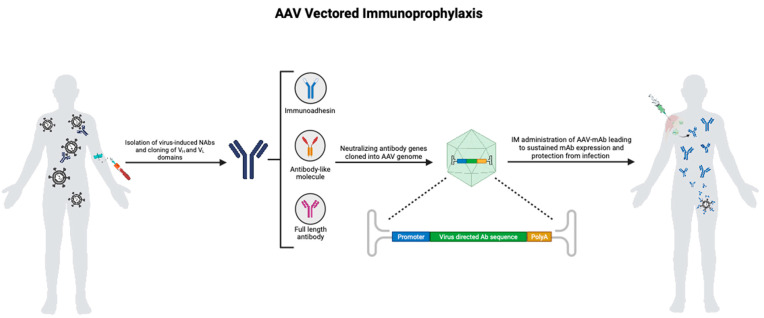
Immunoprotection by AAV-vectored immunoprophylaxis (AAV VIP): Infection of a healthy person with a virus triggers the production of antibodies as part of the host immune response. B cells are harvested from the convalesced individual, and potent neutralizing antibody clones are isolated so that their antibody genes can be cloned and sequenced. Subsequently, the variable-heavy and variable-light chains of neutralizing monoclonal antibodies (mAbs) are cloned into an AAV vector. AAV vectors engineered to express the desired mAbs are manufactured and administered intramuscularly. The transduced muscle cells then secrete these mAbs into the bloodstream, enabling them to circulate throughout the body, providing the host with durable and comprehensive protection against the target pathogen.

An alternative approach to passive immunization that is currently being explored involves the manipulation of gene therapy platforms to deliver highly potent and broadly neutralizing monoclonal antibodies (mAbs) as a one-time treatment. This is achieved by the transduction of long-lived cells with a viral vector engineered to express the heavy- and light-chain domains of an mAb [4]. These domains are separated by a foot-and-mouth disease virus 2A self-cleaving peptide sequence containing a furin cleavage site at the amino terminus to ensure optimal processing [5]. The singular polyprotein produced in vivo should be indistinguishable from those produced by the endogenous immune system [6]. Overall, the desired outcome is rapid and robust expression of a pathogen-specific full-length mAb that will accumulate to therapeutic levels, thereby circumventing the transient nature of traditional passive immunization strategies. This platform has since been called vectored immunoprophylaxis (VIP)—a term coined by David Baltimore and colleagues, who first evaluated this approach as a means to protect against HIV infections [4] (Figure 1).

Adeno-associated virus (AAV) vectors are the current gene delivery vehicle of choice for VIP, because they lack pathogenicity, possess low-level genome integration, target tissues such as the muscle and liver that are ideal for mAb production and secretion, and boast an established record of high-efficiency in vivo gene transfer in a variety of animal models [7,8,9]. There are currently three FDA-approved AAV gene therapies for the treatment of non-infectious diseases on the market, including Luxturna (2017), Zolgensma (2019), and Hemgenix (2022), with several more in the pipeline to be approved in the coming years [10]. When delivered to post-mitotic tissues such as skeletal muscle, in which cells turn over slowly, AAV vector genomes assume the form of high-molecular-weight episomal concatemers that direct transgene expression, often for the lifetime of the cell [11,12]. Based on these attributes, along with the well-established safety profile of AAVs, much research has gone into the evaluation of AAV VIP for establishing long-term expression of therapeutic mAbs directly in vivo.

The VIP system was initially developed as a strategy to establish engineered immunity against HIV [4]. The efficacy of AAV-mediated broadly neutralizing mAb (bNAb) expression was first evaluated in humanized mouse models [4]. These studies not only demonstrated that the AAV VIP system could protect against systemic and mucosal HIV infection, but they also established that AAV-expressed mAbs can persist in circulation at therapeutic levels for the lifetime of the host [4,13]. Follow-up studies in nonhuman primates (NHPs) further demonstrated the effectiveness of this platform, establishing that AAV VIP could protect primates from infection with simian immunodeficiency virus (SIV) [2,14,15,16,17]. Presently, two phase I human clinical trials aimed to evaluate AAV VIP in the context of HIV protection via the delivery of anti-HIV envelope bNAbs [18,19]. Concurrently, the concept has been adopted for the prevention of a variety of other infectious diseases, many of which lack effective vaccines. Indeed, AAV-VIP-mediated protection against malaria, respiratory syncytial virus, influenza virus, Ebola virus, Marburg virus, and neonatal herpes simplex virus, among others, has been achieved in a variety of animal models [20,21,22,23,24,25].

Although animal models such as mice, ferrets, and sheep have demonstrated long-lasting expression of pathogen-specific mAbs delivered with AAV VIP that are capable of conferring protection from viral challenges, translation to higher-order primates is a critical next step [22,24,26,27]. The most representative model for the study of human infectious diseases is the NHP model, due to its close phylogenetic relationship to humans, highly similar immune system, and comparable biology and mucosal tissue physiology [28,29]. However, the antibody persistence, localization, and protection against infection conferred by the AAV VIP platform have not been widely characterized in NHPs, and the immunogenicity of human mAbs in primates remains an important limitation of this model system [15]. Here, we summarize the advancements that have been made evaluating the AAV VIP platform in NHPs, as well as highlighting some important considerations for mAb design in the context of NHP models (see Table 1 for a summary of AAV VIP studies in NHPs). Additionally, we highlight key challenges and gaps that need to be addressed to fully evaluate the clinical potential of AAV VIP as an alternative prophylactic platform to enable long-term passive immunity.

## 2. AAV VIP in the NHP Model

An early proof-of-concept study for the AAV VIP platform, conducted by Lewis et al., demonstrated that mice injected with a recombinant AAV vector encoding the HIV bNAb b12 could produce authentic, biologically active IgG1-b12 that was detectable in sera for up to six months [34]. This study was one of the first to achieve sustained mAb transgene expression without requiring ex vivo transduction or repeated infusions [14]. Follow-up studies aimed to further evaluate the AAV VIP platform using a higher-order animal model to better facilitate clinical translation. As mentioned earlier, the AAV VIP platform was originally developed for the prevention/treatment of HIV [4]. Consequentially, research has progressed more rapidly for this indication. Currently, the only infectious disease for which AAV VIP has been evaluated in an NHP model is HIV (and simian human immunodeficiency virus (SIV) as a surrogate for HIV) [2,14,15,16,17,30,31,32]. The following sections will review the most prominent HIV AAV VIP studies conducted in NHPs, with an emphasis on vector and study design.

### 2.1. AAV-Mediated Expression of Immunoadhesins

Johnson and colleagues were the first group to evaluate the AAV VIP platform in an NHP model [14]. At the time when these studies were initiated, human bNAbs were rare and difficult to induce; therefore, they were not a feasible transgene candidate [14]. Instead, their investigations focused on the design and delivery of immunoadhesin molecules using the AAV VIP platform. Immunoadhesins are chimeric antibody-like molecules consisting of the functional domain of a receptor or adhesion molecule bound to the Fc region of an Ig molecule [35].

SIV glycoprotein 120 (gp120)-specific fragment antigen-binding (Fab) molecular clones isolated from an infected macaque (346–16 h and 342–23 h) were employed in the design of the chimeric molecules. To generate single-chain (sc) FC-Fc immunoadhesins, the CH2-CH3 domain of the rhesus IgG2 antibody was fused to the sc variable fragment (scFv) of mAbs 4L6 and 5L7. A CD4 immunoadhesin was also designed by fusing Ig-like domains 1 and 2 of CD4 to the Fc fragment of rhesus IgG2 (referred to as N4). Both 4L6 and 5L7 were expressed from a self-complementary AAV (scAAV) genome, whereas N4 was expressed from a single-stranded AAV (ssAAV) genome. All three transgenes were under the control of a CMV promoter and packaged into an AAV1 vector. Groups of three rhesus macaques were administered a dose of 2 × 10^13^ vector genomes (vg) per animal, split into four deep intramuscular (IM) injections of 0.75 mL (two injections per quadriceps). At four weeks post-AAV administration, immediately prior to challenge with SIV, transgene expression in macaque sera varied significantly between and within the experimental groups. The 4L6-immunized monkeys had the highest transgene expression levels, reaching concentrations between 100 and 190 μg/mL. Similar levels were measured in two monkeys from the 5L7 group (05C002 and 05C053), expressing 40 μg/mL and 175 μg/mL, respectively. Interestingly, although the third 5L7 monkey (05C004) expressed around 50 μg/mL of transgene two weeks after AAV administration, its levels returned to baseline by the time of challenge. Finally, monkeys in the N4 group were noted to have significantly lower immunoadhesin expression at the time of challenge, ranging from 3 to 10 μg/mL, potentially due to the alternative design of the AAV vector. The wide range in expression levels appeared to coincide with the appearance of anti-transgene antibodies, defined as the anti-drug antibody response (ADA). Upon intravenous (IV) challenge with SIVmac316, 6 of the 12 treated animals resisted infection: 3 macaques from the 4L7 group, 2 from the N4 group, and 1 from the 5L7 group. Subsequent serum analysis further demonstrated measurable serum transgene expression in several of the experimental animals for up to one-year post-challenge.

This study was groundbreaking because it represented one of the first successful examples of an AAV vector directing long-term expression of a biologically active monoclonal antibody in an NHP model. However, the apparent transgene immunogenicity observed in ~33% of treated macaques, coupled with the modest protection against SIVmac316, demonstrated the need for further optimization before any clinical translation of the AAV VIP platform.

### 2.2. AAV-Mediated Expression of Antibody-like Molecules

Due to the partial protection conferred by immunoadhesin molecules, Gardner at al. took an alternative approach to transgene design [30]. CD4 and its co-receptor binding sites are known to be the most conserved regions on the HIV-1 envelope (Env) glycoprotein [36,37,38]. The immunoadhesin form of CD4, CD4-Ig (CD4 domains 1 and 2 fused to the Fc domain of human IgG1), has been well characterized and is known to neutralize most Env isolates. However, it has a low affinity for Env and possesses the ability to promote infection. The mimetic form of CCR5, CCR5mim1, is another molecule that is capable of binding Env with high specificity and affinity and is capable of targeting both CCR5- and CXCR4-dependent Env proteins. It was therefore reasoned that by fusing the CCR5mim1 sulfopeptide to the carboxyl terminus of a CD4-Ig molecule, the resultant fusion protein would be capable of binding to Env cooperatively and with the highest avidity of the three molecules [30].

To evaluate whether this molecule, known as eCD4-Ig, would function as an antibody-like entry inhibitor in NHPs, the fusion protein design was modified to incorporate Ig-like domains 1 and 2 of a rhesus CD4 molecule, with the addition of an Ile39Asn mutation to preserve the high potency of the human CD4 molecule for HIV isolates [39]. Furthermore, to minimize immunogenicity, the Fc domain of a rhesus IgG2 was used, since it is believed to bind Fc receptors and complement less readily than IgG1 [30,40]. The resultant rh-eCD4-IgG2^I39N,mim2^ molecule (described hereafter as rh-eCD4-Ig) was cloned into an ssAAV1 vector and delivered at a dose of 2 × 10^13^ vg/animal through two deep 0.5 mL IM injections (one per quadriceps). As a part of the two injections, 0.5 × 10^13^ vg/animal of AAV1-expressing rhesus tyrosine-protein sulfotransferase 2 (AAV-rh-TPST2) was co-administered to promote rh-eCD4-Ig sulfation. Eight weeks after AAV inoculation, the treated macaques, along with four age- and gender-matched controls, were challenged IV with 2 pg p27 SHIV-AD8-EO. Uninfected animals received repeated escalating challenges over several weeks, reaching 800 pg p27 34 weeks post-treatment. Upon challenges with SHIV-AD8, all treated macaques resisted infection, as evidenced by the lack of viral RNA in their blood, whereas control animals succumbed to infection. Serum transgene expression persisted for more than 50 weeks after AAV administration at concentrations that were well tolerated and protective against successive challenges, with expression stabilizing between 17 and 77 μg/mL over the last 10 weeks of the experiment. Although modest ADA responses were observed in two of the four treated macaques, this returned to baseline by week 37. Subsequent challenges with SHIV-AD8 that were 8 and 16 times higher than the 50% animal infectious dose (AID_50_) were administered at 52- and 56-weeks post-treatment, respectively. Once again, all four experimental macaques were able to resist infection, as evidenced by the lack of viral RNA in their blood. At the conclusion of the study, all inoculated macaques resisted infection and were significantly protected from the SHIV-AD8-EO challenges. Although the doses used were greater than what would be commonly experienced during a human transmission event, mucosal challenges were not evaluated at the time.

A follow-up study conducted by the same group evaluated the protective abilities of rh-eCD4-Ig against the more infectious SIVmac239 [17]. Using a similar experimental design, four macaques received a 2 × 10^13^ vg/animal dose of AAV1-rh-eCD4-Ig co-administered with a 0.5 × 10^13^ vg/animal dose of AAV1-TPST2 through IM injections of the quadriceps. Serum transgene expression levels peaked at 13–44 μg/mL four to five weeks after AAV inoculation and plateaued between 3 and 18 μg/mL. The ADA response, which was primarily against the sulfopeptide, was notably higher in all four experimental animals compared to what was observed in the previous study. However, the ADA response returned to baseline 14 weeks after AAV administration in three of the four animals. Following a 22-week period after AAV administration, monkeys were intravenously challenged with 20 pg p27 SIVmac239. Animals that remained uninfected were rechallenged every four weeks with twice the previous dose. Although all treated monkeys resisted infection at 40 pg p27—the dose at which all control animals became infected—one treated monkey (rh2448), with the lowest rh-eCD4-Ig expression and highest ADA response, became infected shortly after at 80 pg p27. With repeated escalating challenges, the remainder of the treated animals succumbed to infection at 8, 16, and 32 times the dose that infected control animals, 42, 46, and 50 weeks after AAV inoculation, respectively.

These studies demonstrated that AAV-mediated expression of rh-eCD4-Ig can persist for over a year at serum concentrations sufficient to protect against high doses of SHIV-AD8-EO. Additionally, they showed robust protection against SHIV-AD8 and SIVmac239—two very divergent strains of HIV. These proof-of-concept studies also highlighted the potential of AAV VIP for neutralizing a variety of genetically distinct HIV strains, and they proved that sustained AAV-mediated expression of antibody-like molecules is possible despite the occurrence of low-to-moderate ADA responses.

### 2.3. AAV-Mediated Expression of Non-Native Full-Length Antibodies

Similar to antibody-like molecules, numerous studies have evaluated AAV-mediated expression of full-length antibodies as an immunoprophylaxis against a wide range of viral diseases, as well as some parasitic and bacterial pathogens [6,20,41,42]. These studies, which were primarily conducted in rodent models, facilitated the initial optimization of several key aspects of the AAV VIP platform, including mAb and isotype selection, vector design, and mode of administration. In the meantime, several promising bNAbs isolated from HIV-infected individuals have been characterized and validated in small-animal models [43]. These full-length antibodies proved to be highly potent, in many cases neutralizing HIV more efficiently than immunoadhesins, and making them an attractive therapeutic modality to explore in the context of AAV VIP in NHPs [44].

Saunders and colleagues were among the first groups to evaluate AAV-mediated expression of full-length mAbs in an NHP model [15]. In their study, the human bNAb VRC07 (huVRC07) was simianized (simVRC07) by combining simian IgG1 constant regions with macaque framework variable regions containing minimal transplanted huVRC07 genes. Using an AAV8 vector, the simVRC07 gene was administered to four monkeys at a dose of 1 × 10^13^ vg/animal by a singular 1 mL IM injection to the quadriceps. Serum mAb expression peaked between two and four weeks after AAV treatment at 2.5–7.7 μg/mL and fell below detectable levels by week nine, with strong ADA responses detected in all animals, mainly directed at the huVRC07 residues within the simVRC07 construct. The group followed up these experiments with a similarly designed study, this time with the addition of the immunosuppressive agent cyclosporine (CsA). This immunosuppressant has previously been shown to permit AAV-mediated expression of human factor IX in macaques [45]. In this trial, six monkeys were intravenously infused with 5 mg/kg CsA eight and nine days prior to AAV treatment. Six days prior to treatment, the group switched to 15–30 mg/kg oral CsA treatments every other day, continuing out to day 28 after AAV vector administration. The control group of macaques received a CsA dosing regimen identical to that of the treatment group and were administered with an AAV8 vector encoding a simianized non-HIV antibody (AAV8-control IgG). The addition of an immunosuppression protocol increased simVRC07 serum expression levels, peaking around 38 μg/mL in the first three weeks. To evaluate the protective efficacy of AAV8-simVRC07, CsA treatments were halted 11 days prior to mucosal challenge. During this period, a significant drop in transgene expression was noted, with simVRC07 expression falling below detectable levels in three out of six experimental animals. At five and a half weeks after AAV treatment, all monkeys were intrarectally challenged with 12,800 50% tissue culture infectious doses (TCID_50_)/mL of CCR5-tropic SHIV-BaLP4. All five control monkeys became infected, along with two monkeys (A11E035 and A11E045) from the treatment group who had the lowest peak and day-of-challenge transgene expression. The remaining four macaques treated with AAV expressing simVRC07 remained uninfected, demonstrating sufficient protective efficacy. This study was the first demonstration of a full-length antibody being expressed via AAV-mediated gene transfer in an NHP model. It was also the first study to achieve protection against mucosal infection in a macaque model of HIV infection following gene transfer of bNAbs. Additionally, these results showed that alternative capsids—in this case, AAV8—could be used to successfully deliver mAb transgenes in NHPs. However, AAV-mediated expression of non-native full-length mAbs resulted in stronger ADA responses than were previously observed in NHP studies where AAV1 was used to express simian-derived immunoadhesins.

In a landmark study by Fuhcs et al., full-length rhesus IgG1 anti-SIV mAbs 5L7 and 4L6, derived from the bone marrow of SIV-infected rhesus macaques and expressed under the control of a CMV promoter, were simian-codon-optimized and packaged into an AAV1 capsid [16]. The main goals of the study were to compare transgene expression efficiency between ssAAV and scAAV vectors, as well as to evaluate whether the ADA response seen in the immunoadhesin versions of 5L7 and 4L6 expressed from AAV in NHPs could be alleviated with the use of a full-length antibody. In their first experiment, six monkeys were equally divided into two groups to evaluate 5L7 expression. One group received 1.6 × 10^13^ vg/animal ssAAV expressing both heavy and light chains of the 5L7 IgG1, while the other group received 0.8 × 10^13^ vg/animal scAAV1 expressing the 5L7 IgG1 heavy chain and 0.8 × 10^13^ vg/animal scAAV1 expressing the kappa light chain of 5L7 IgG1. The vector constructs were delivered via four deep IM injections, with each animal receiving two 0.5 mL injections per quadriceps. Significant monkey-to-monkey variation in transgene expression was observed, ranging from 1 to 270 μg/mL, and expression was correlated directly with the magnitude of the ADA response. Furthermore, the results suggested no significant differences in transgene expression between the ssAAV and scAAV groups. Although transgene expression in both 5L7 groups remained relatively low, one NHP (84-05) in the ssAAV1-5L7 group demonstrated robust and sustained serum mAb concentrations of 270 μg/mL. This NHP was monitored for over six years and successfully maintained serum concentrations of mAb 5L7 ranging between 240 and 350 μg/mL [46]. The same research group followed up with another experiment in which six monkeys were inoculated with 2.5 × 10^13^ vg/animal ssAAV1-4L6 IgG1 through four deep intramuscular injections [16]. Clear ADA responses were recorded in all six animals. At the time of challenge, mAb 4L6 expression ranged between 1 and 38 μg/mL in four of the six macaques, with the other two falling below the detectable limit. It should be noted that in both experiments the ADA response was directed primarily against the variable domains and not the constant domains of the IgG molecules. The 5L7- and 4L6-treated groups were subsequently challenged with 1x AID_50_ SIVmac239 44 and 14 weeks after AAV treatment, respectively. Following six repeated incremental IV challenges, only one monkey (84-05) remained uninfected. While the results of this study demonstrate that it is possible to mediate sustained mAb expression at therapeutic concentrations from an AAV vector, they also underscore the need to better understand and mitigate the ADA response to fully realize the potential of the AAV VIP platform.

A subsequent study from Ronald Desrosiers’ group investigated whether AAV-delivered mAbs could lead to long-term virologic suppression in macaques chronically infected with simian–human immunodeficiency virus (SHIV) [32]. In this study, HIV bNAbs 10E8, 3BNC117, and 10-107 were engineered to encode the IgG1 Fc domain from rhesus macaques to maintain effector functions. An additional “LS mutation” was introduced into the Fc portion of each antibody to increase their half-lives [47,48]. Target sequences for specific miRNAs, such as mir-142-3p, which is highly expressed in antigen-presenting cells (APCs) [49,50,51], were encoded into the AAV transgene cassette. This genome modification promotes the post-transcriptional cleavage of transgene mRNAs inadvertently expressed in APCs, thereby mitigating potential off-target expression of mAbs that could contribute to ADA responses. A group of four macaques were challenged by IV injection with SHIV-AD8eo, a viral strain capable of maintaining chronic-phase viremia and inducing progressive CD4^+^ cell depletion and acquired immunodeficiency syndrome (AIDS) [32,52]. The infected NHPs were administered with three different recombinant AAV-mAbs packaged into an AAV1 vector at a dose of 2 × 10^12^ vg/kg through two 0.75 mL IM injections to the right quadriceps (10–1074), left quadriceps (3BNC117), and right and left deltoid (10E8) 86 weeks after SHIV-AD8eo infection. While serum expression of modified human bNAbs 10E8, 3BNC117, and 10-1074 was hampered by the ADA response, one macaque (rh2438), termed the “Miami monkey”, was able to maintain 50–150 μg/mL of 3BNC117 and 10–1074 in the blood for over two years. This AAV-mediated mAb expression resulted in complete virologic suppression of SHIV infection for 38 successive measurements over three years, without any repeated administrations or antiviral therapies. To extend the success of the “Miami monkey”, the researchers infected an additional 12 rhesus macaques with SHIV-AD8eo. Following a 30–36-week period, the macaques were evenly split into a “bi-group” and a “quad-group”. The “bi-group” received 2 × 10^12^ vg/kg AAV8-3BNC117 and AAV8-10-1074 through four 0.75 mL IM injections to the left and right quadriceps, respectively. The “quad-group” received 2 × 10^12^ vg/kg of mAbs N6, 35022, PGT145, and PGT128 packaged into an AAV8 vector as eight 0.75 mL IM injections to the left quadriceps, right quadriceps, left deltoid and bicep, and right deltoid and bicep, respectively. A subsequent AAV1 booster inoculation encoding the same mAbs received in the initial AAV administration was administered to all 12 macaques 54–60 weeks after SHIV infection, at a dose of 1 × 10^12^ vg/kg. The findings from this follow-up study did not indicate boosters to be effective, and strong ADA responses were observed in all macaques. Although a dip in viral loads was apparent in 9 of the 12 treated animals, this virologic suppression was only transient. Furthermore, the booster appeared to exert little-to-no effect on the viral loads. Nevertheless, this pivotal study provided proof of concept that the AAV VIP platform could eventually afford a “functional cure” for HIV, provided ADA responses can be prevented.

Finally, a recent study by Michael Farzan’s group evaluated the impact of antibody isotype on the longevity and ADA response to AAV-mediated mAb expression [31]. The majority of AAV-expressed mAbs have been of the IgG1 isotype, in large part due to the longer half-life and beneficial Fc effector functions associated with this isotype; however, mAbs of the IgG1 isotype have also been associated with significant ADA responses in NHPs [15,16,32]. To determine whether Ig subtype has an influence on immunogenicity, 12 macaques were divided into four equal groups and inoculated with 1 × 10^13^ vg of AAV1 expressing rhesus IgG1 or IgG2 isotypes of rhesusized human anti-HIV bNAbs 3BNC117 or NIH45-46, along with the same amount of vector encoding 10-1074 or PGT121 of the same isotype. Each group received two AAV mAbs of the same isotype through two 0.5 mL deep IM injections to the left and right quadriceps. Serum transgene levels peaked two to four weeks post-treatment between 3 and 69 μg/mL but dropped precipitously concurrent with the appearance of ADA responses. However, the authors found that bNAb combinations with IgG2-Fc domains elicited significantly lower ADA responses than their IgG1 counterparts. They also observed better protection from two SHIV-AD8 challenges 8 weeks (100 pg p27) and 10 weeks (200 pg p27) post-treatment in macaques administered with AAV1 expressing IgG2-isotyped bNAbs, compared to those administered with IgG1-isotyped bNAbs. These data suggest that AAV expression of mAbs with IgG2-Fc domains might be less immunogenic than that of those with IgG1 Fc domains, and they critically highlight ADA responses as an important barrier to the use of AAV for in vivo expression of bNAbs, and potentially for other types of mAbs. Of course, the use of IgG2 isotypes will only be possible in scenarios where antibody effector functions are unnecessary and/or less relevant.

### 2.4. AAV-Mediated Expression of Native Full-Length Antibodies

Due to the significant ADA response generated towards engineered human bNAbs, attention has been directed towards the delivery of native, autologous anti-SIV neutralizing antibodies in NHP models. Welles et al. designed a fully rhesus system wherein native anti-SIV Env-specific mAbs (ITS01 and ITS06.02) were administered to rhesus macaques using an AAV8 vector [2]. In a pilot study, 1 × 10^13^ vg each of AAV8-ITS01 and AAV8-ITS06.2 were IM administered to both quadriceps of six monkeys, leading to stable transgene expression plateauing around 21.2 and 8.6 μg/mL for each mAb, respectively. Vector dosage was evaluated through the administration of AAV8-ITS01 or AAV8-ITS06.2 at a high (2 × 10^13^ vg), medium (2 × 10^12^ vg), or low (2 × 10^11^ vg) dose. The high-dose group produced mildly higher transgene expression levels that became indistinguishable from those of the medium-dose group over time, suggesting that an injection site saturation point is reached around the medium dose. Using the mid-range dose of 2 × 10^12^ vg AAV8, the number of injection sites was evaluated. Intramuscular injections were distributed over one, two, four, or eight injection sites in the right quadriceps, both quadriceps, both quads and gastrocnemius, or quads, gastrocs, biceps brachii, and deltoids, respectively. It should be noted that the peak serum mAb expression followed a near-linear dependence on the number of injections, with the highest mAb expression levels detected in NHPs that received the AAV dose spread out over eight injection sites. While these differences waned by week 24, the two NHPs receiving eight injections exhibited more consistent high-level mAb expression. These data suggest that a single AAV bolus injection may saturate the injection site when administered at high doses, and that improved mAb expression from AAV might be achieved by using a delivery device that can target multiple distinct injection sites, rather than by increasing the AAV dose.

Finally, a mucosal challenge model was evaluated, but unlike previous studies, the challenge strain did not consist of an SIV/SHIV but rather SIVsmE660, a native rhesus strain of the virus. An initial group of six and a follow-up group of four macaques were administered 5 × 10^12^ vg/animal of AAV8-ITS01 and ITS06.2. At the time of intrarectal challenge, serum mAb expression ranged between 10 and 100 μg/mL. Early investigations of immunoadhesins and Fc-fusion proteins had presented low-to-moderate ADA responses; however, later investigations of human bNAbs resulted in significantly greater immunogenic responses. One of the main aims of the study was to evaluate the hypothesized link between an mAb transgene’s degree of divergence from germline and the strength of the resultant ADA response. In agreement with the generalized hypothesis, minimal ADA responses were noted in 20% of the AAV8-ITS01- and AAV8-ITS06.02-treated animals. These results were a significant improvement from prior studies that reported ADA responses in 50–100% of experimental animals [15,16,31,32]. The results of this study showed that AAV8-mediated expression of mAbs ITS01 + ITS06.2 was able to decrease the rate of SIVsmE660 infection by 90% compared to historic controls, and by 75% using contemporaneous controls. These results suggest that it may be possible to avoid the induction of ADA responses if autologous, naturally arising mAbs are expressed from AAV, as would be done clinically.

A recent follow-up study carried out by Gardner et al. aimed to further optimize the AAV VIP delivery system [33]. Many studies examining the expression of HIV, Ebola virus, and Marburg virus antibodies have all included an F2A peptide sequence; however, previous work has indicated that porcine teschovirus-1 2A (P2A) and *Thosea asigna virus* 2A (T2A) peptides might be more efficient alternatives [5,53]. The researchers first evaluated transgene cassette design, comparing the efficiencies of P2A, F2A, and T2A self-cleaving peptides expressed within the sequence of the anti-SIV antibody ITS01 by an AAV9 vector in mice. Robust expression (>100 μg/mL) was observed in all three experimental groups by the end of the study, yet at eight weeks post-treatment a twofold increase in ITS01 expression was observed in the P2A group (160–402 μg/mL) when compared to the other two experimental groups. Moving forward with the P2A peptide cassette design, the group then focused on the evaluation of AAV capsid serotype in the context of enhancing transgene expression and minimizing ADA responses. Five capsids—AAV1, AAV8, AAV9, and the engineered capsids AAV-NP22 and AAV-KP1—were designed to express the ITS01 bNAb and delivered to groups of rhesus macaques at a dose of 2.5 × 10^12^ vg/kg by eight deep IM injections, including two injections per quadriceps, one injection per bicep, and one injection per deltoid [54,55]. Following successive serum collections, it was determined that the AAV9 capsid conferred the greatest average ITS01 expression, averaging around 224–302 μg/mL 10 weeks post-treatment. The AAV1 capsid represented the second-greatest accumulation of serum transgene levels, at 216–243 μg/mL. All other serotypes resulted in serum transgene levels below 100 μg/mL. Encouragingly, an ADA response was only detected in 31.6% of NHPs, likely due to the minimal degree of antibody mutation, since ITS01 is only ~5–6% divergent from its closest germline precursor antibody in the macaque repertoire [2]. The findings of this study support the notion that the number of injection sites used may be essential in targeting a greater population of muscle cells and thereby increasing transgene expression. Furthermore, it may be important to evaluate different self-cleaving peptides to maximize the transgene expression of different mAbs. Finally, although AAV1 has historically been the capsid of choice for AAV VIP, this study highlighted AAV9 as a viable alternative.

## 3. AAV VIP in Other Large-Animal Models

In addition to HIV, the AAV VIP platform has been shown to protect against a wide range of infectious diseases, including influenza, malaria, respiratory syncytial virus, Ebola virus, and Marburg virus, to name a few [19,20,22,23,25]. Marburg virus (MARV), in particular, has been designated a priority disease by the WHO because it possesses significant epidemic potential and lacks an approved vaccine or countermeasure [41]. Thus, AAV VIP has been explored as an alternative prophylaxis for this high-consequence pathogen.

**Figure 2:**
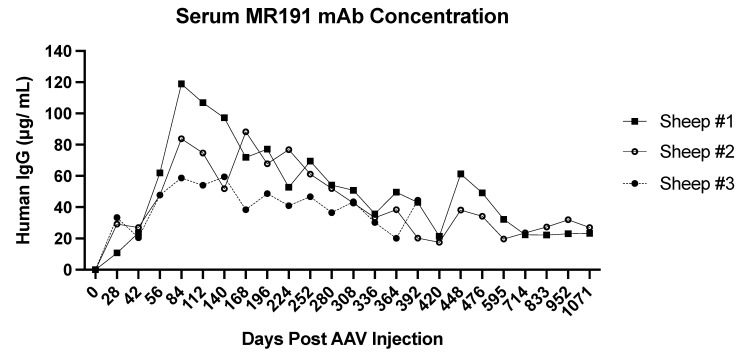
Long-term monitoring of AAV6.2FF-mediated expression of mAb MR191 in sheep serum following intramuscular administration of 5 × 10^12^ vg/kg of AAV6.2FF-MR191.

Our group has demonstrated that AAV6.2FF-mediated expression of the anti-MARV GP mAb, MR191, as a human IgG1 molecule, protects mice [24] and guinea pigs (Rghei et al., under review) from lethal challenges with MA-MARV and GPA-MARV, respectively. Additionally, we have evaluated the duration of AAV6.2FF-mediated MR191 mAb expression in sheep as a representative outbred large-animal model. Two-week-old lambs administered IM with 5 × 10^12^ vg/kg of AAV6.2FF-MR191 yielded serum human IgG (hIgG) expression that was sustained for over 460 days, concomitant with low levels of anti-drug antibodies. Serum hIgG concentrations peaked between 67 and 119 μg/mL in the first 126 days [24], with sustained average serum concentrations of ~37 μg/mL for >450 days. Based on challenge studies in mice, this concentration of mAb expression is within the therapeutic range for protection against MARV infection [24]. Blood collections were continued for almost three years, at which point the two remaining animals in the study had serum MR191 mAb concentrations of 23.22 μg/mL and 26.99 μg/mL (Figure 2).

Another goal of this study was to evaluate the ADA response in sheep. Although low-level anti-human IgG responses were detected, they remained low throughout the study and did not appear to negatively impact transgene expression [24]. The duration of AAV-mediated mAb expression and the low-level ADA responses observed in this study suggest that the nature of the mAb and the AAV capsid play an important role in impacting the efficacy of the AAV VIP platform. Future studies will evaluate the ability of AAV6.2FF-mediated expression of MR191 to protect NHPs from a lethal challenge with MARV.

## 4. AAV VIP in Human Clinical Trials

The first clinical trial evaluating AAV VIP for the prevention of HIV infection used AAV1 expressing HIV bNAb PG9 [18,56,57]. In this trial, the heavy and light chains of PG9 were under the control of a CMV and EF1alpha promoter, respectively [58]. Healthy, non-HIV-infected men were administered IM with AAV1-PG9 at doses ranging from 4 × 10^12^ vg to 1.2 × 10^14^ vg. Muscle biopsies showed detectable PG9 mRNA expression, as well as IgG expression within muscle cells and the adjacent interstitium via immunohistochemical analysis. However, no PG9 was detectable in the serum by ELISA, although it is possible that PG9 might have been expressed at levels below the assay’s limit of detection (2.5 μg/mL). Notably, ADA responses were detected in many of the participants, mainly in the high-dose group, which likely limited the amount of PG9 in circulation [18]. Lack of PG9 in the serum indicated that further optimization of the mAb expression cassette design, mAb selection, AAV capsid serotype, and dosing is required.

In a subsequent phase I clinical trial assessing the expression of AAV-bNAbs, the safety and efficacy of AAV8-VRC07 was investigated in HIV-infected adults [19]. The vector genome design in this trial differed from the first trial in that the heavy and light chain domains of VRC07 were expressed as a monocistronic mRNA from the muscle-optimized CASI promoter. Upon translation, the heavy and light chains were cleaved into two separate proteins through the inclusion of an F2A self-cleaving peptide. The administration of AAV8-VRC07 was performed through intramuscular injection, with doses ranging from 5 × 10^10^ to 2.5 × 10^12^ vg/kg. After AAV injection, three out of eight participants achieved serum VRC07 concentrations exceeding >1 μg/mL. Remarkably, these participants maintained this level of serum VRC07 for up to three years post AAV administration. However, non-idiotypic ADA responses, directed towards the Fab portion of the VRC07 transgene, were observed in three participants. Of these three individuals, two experienced a decline in serum VRC07 levels due to these immune responses. Additionally, a dose dependent anti-AAV8 capsid immune response was reported in all participants following vector administration. Importantly, this groundbreaking study demonstrated, for the first time, that AAV has the capacity to induce sustained production of bNAbs in HIV-infected individuals following intramuscular administration.

## 5. Conclusions

The aim of the VIP platform is to confer long-term expression of protective mAbs to facilitate immunity against infectious diseases. Although mice are an easily accessible, cost-effective model system for the evaluation of AAV mAb expression, they are not ideal for clinical translation. As seen with many of the AAV VIP anti-HIV mAb studies, translation from mice to NHPs has been plagued by low or transient mAb expression, mainly in conjunction with the appearance of strong ADA responses [14,15,16,31,32]. In NHP studies, the anti-transgene antibody response is primarily directed towards the variable regions of HIV bNAbs [16,31,32]. These bNAbs have extensive somatic hypermutations in their variable domains, with long variable-heavy-chain complementary-determining regions that render them highly diverse from germline [31]. It might be reasoned that these characteristics could make HIV bNAbs more immunogenic than other mAbs isolated from human survivors of acute viral infections. Additional research evaluating the AAV VIP system in NHP models for other infectious diseases will be required to better characterize and mitigate the ADA response.

AAV VIP studies in NHPs have highlighted the need for further refinements to the platform. Future research should focus on identifying optimal regulatory [59] and immune-suppressing sequences [60] to incorporate into the AAV genome, as well as evaluating different capsid and antibody sequences [41] and investigating the use of different muscle-specific promoters. More research should evaluate the impact of co-administering transduction-enhancing agents such as rapamycin [61,62] and doxorubicin [63,64]. Finally, improvements to AAV manufacturing are needed to bring the cost of production down.

The AAV VIP system has the potential to provide protection against a host of infectious diseases, given that AAV mAbs retain their function, achieve mucosal translocation, and yield stable long-term expression. This passive immunization approach is an ideal substitute for passive infusions, as it relies on a one-time treatment. To develop adequate infrastructure to allow for the AAV VIP platform to be scaled to a level capable of combating pandemic diseases, a substantial commitment from governments and industry will be required.

## Figures and Tables

**Table 1:** Summary of AAV VIP studies conducted in NHP models to date.

Transgene Class	Transgene Species Origin	Transgene Name	Transgene Function	Antibody Isotype	AAV Capsid Serotype	Vector Modification	AAV Vector Dose	Serum Concentration of Transgene *	ADA Response	Challenge Virus	Pre-Screening of NHPs **	Immune Modulation	Reference
Immunoadhesin	Simian	4L6	binds gp120	rhesus IgG2 Fc	scAAV1	NA	2 × 10^13^ vg	100–190 μg/mL time of challenge	0 of 3 macaques	SIVmac316 IV	SIV negative	NA	[14]
		5L7						0–175 μg/mL time of challenge	2 of 3 macaque				
		N4	binds CD4 receptor on Env		ssAAV1			3–10 μg/mL time of challenge	1 of 3 macaque				
Fc-Fusion Protein	Simian-Human chimera	rh-eCD4-IgG2^I39N,mim2^	binds CD4 receptor on Env	rhesus CD4 domain 1 & 2 and IgG2 Fc & hinge	ssAAV1	Ile39 mutation in CD4 domain	2 × 10^13^ gc	13–44 μg/mL	all 4 macque	SIVmac239 IV	AAV1 negative SIV negative	Co-administered 0.5 × 10^13^ gc AAV1-TPST2	[17]
	Simian-Human chimera	rh-eCD4-IgG2^I39N,mim2^	binds CD4 receptor on Env	rhesus CD4 domain 1 & 2 and IgG2 Fc & hinge	ssAAV1	Ile39 mutation in CD4 domain	2.5 × 10^13^ gc	plateau 17–77 μg/mL for 40 weeks	2 of 4 macaque	SHIV-AD8-EO IV	AAV1 negative	Co-administered 0.5 × 10^13^ gc AAV1-TPST2	[30]
Non-native full length antibodies	Human	VRC07	CD4 receptor on Env	simianized IgG1	AAV8	somatic mutation of framework CDR region from human to macaque	1 × 10^13^ vg	2.5–7.7 μg/mL	all 4 macaque	NA	AAV8 negative	NA	[15]
								38.12 μg/mL	3 of 6 macaque	CCR5-tropic SHIV-BaLP4 REC		CsA infusion 5 mg/kg; oral 15–30 mg/kg	
	Simian-Simian chimera	5L7	Env gp140	rhesus IgG1 CH1 and CL domains	scAAV1	NA	2× 8 × 10^12^ vg	20–55 μg/mL	1 of 3 macaque	NA	AAV1 negative SIV negative	NA	[16]
					ssAAV1		1.6 × 10^13^ vg	53–270 μg/mL	2 of 3 macaque	SIVmac239 IV			
		4L6			ssAAV1		2.5 × 10^13^ vg	45–150 μg/mL	all 6 macaque				
	Human	3BNC117	CD4 receptor on Env	rhesus IgG1 or IgG2 constant regions	AAV1	NA	1 × 10^13^ gc	3–69 μg/mL	all 12 macaque	SHIV-AD8 IV	AAV1 negative SIV negative	NA	[31]
		NIH45–46											
		10-1074	N332 V3-glycan site on Env										
		PGT121											
	Human	10E8	epitope in gp41	rhesus IgG1 constant heavy and light chains	AAV1	“LS” mutation in Fc & mir-142-3p sequence in vector	2 × 10^12^ vg/kg	2–10 μg/mL	all 4 macaque	SHIV-AD8e0 IV	AAV1 negative HIV negative SIV negative	NA	[32]
		3BNC117	CD4 receptor on Env gp120					0–150 μg/mL					
		10-1074	C2 V3 region of gp120					20–200 μg/mL					
		3BNC117	CD4 receptor on Env gp120		AAV8 prime AAV1 boost		2 × 10^12^ vg/kg prime 1 × 10^12^ vg/kg boost	0.9–5.8 μg/mL	all 12 macaques		AAV1 negative AAV8 negative HIV negative	Gammagard 10% liquid (IVIG) 1 g/kg	
		10-1074	C2 V3 region of gp120					0.2–75 μg/mL					
		N6	CD4 receptor on Env					0–13 μg/mL					
		35O22	novel Env epitope					0–8.9 μg/mL					
		PGT128	C2 V3 region of gp120					0–60 μg/mL					
		PGT145	oligomannose glycans					0.2–53 μg/mL					
Native full-length antibodies	Simian	ITS01	binds CD4 receptor on Env	fully native rhesus IgG1	AAV8	NA	1 × 10^13^ gc/animal	21.2 μg/mL	~20% of macaques	SIVsmE660 REC	AAV8 negative	NA	[2]
		ITS06.02	binds variable loop 1					8.6 μg/mL					
		ITS01	binds CD4 receptor on Env				5 × 10^12^ gc/animal	20–100 μg/mL					
		ITS06.02	binds variable loop 1										
	Simian	ITS01	binds CD4 receptor on Env	fully native rhesus IgG1	AAV1	NA	2.5 × 10^12^ vg/kg	216–243 μg/mL	2 of 3 macaque	not a challenge model	screened for pre-existing AAV neutralizing antibodies and grouped accordingly	NA	[33]
					AAV8			>100 μg/mL	3 of 5 macaque				
					AAV9			224–302 μg/mL	0 of 5 macaque				
					AAV-NP22			>100 μg/mL					
					AAV-KP1				1 of 3 macaque				

vg, vector genomes; gc, genome copies; IV, intravenous administration; REC, intrarectal administration; TPST2, tyrosine-protein sulfotransferase 2; CsA, cyclosporine; IVIG, intravenous immunoglobulin. * Transgene serum concentrations were estimated based on graphical interpretation. All ranges are at peak expression except when indicated otherwise. ** All studies covered here used Rhesus macaque of Indian origin.

## Data Availability

Not applicable.
